# Prediction of microvascular invasion of hepatocellular carcinoma: value of volumetric iodine quantification using preoperative dual-energy computed tomography

**DOI:** 10.1186/s40644-020-00338-7

**Published:** 2020-08-18

**Authors:** Taek Min Kim, Jeong Min Lee, Jeong Hee Yoon, Ijin Joo, Sae-Jin Park, Sun Kyung Jeon, Bernhard Schmidt, Sedlmair Martin

**Affiliations:** 1 Department of Radiology, Seoul National University Hospital, Seoul, South Korea; 2 Department of Radiology, Seoul National University College of Medicine, Seoul, South Korea; 3 Institute of Radiation Medicine, Seoul National University Medical Research Center, Seoul, South Korea; 4 Research and development department, Siemens Healthineers, Forchheim, Germany

**Keywords:** Hepatocellular carcinoma, Microvascular invasion, Dual-energy CT, Peritumoral enhancement, Iodine quantification, Iodine concentration

## Abstract

**Background:**

To investigate the potential value of volumetric iodine quantification using preoperative dual-energy computed tomography (DECT) for predicting microvascular invasion (MVI) of hepatocellular carcinoma (HCC).

**Methods:**

This retrospective study included patients with single HCC treated through surgical resection who underwent preoperative DECT. Quantitative DECT features, including normalized iodine concentration (NIC) to the aorta and mixed-energy CT attenuation value in the arterial phase, were three-dimensionally measured for peritumoral and intratumoral regions: (i) layer-by-layer analysis for peritumoral layers (outer layers 1 and 2; numbered in close order from the tumor boundary) and intratumoral layers (inner layers 1 and 2) with 2-mm layer thickness and (ii) volume of interest (VOI)-based analysis with different volume coverage (tumor itself; VOI_O1_, tumor plus outer layer 1; VOI_O2_, tumor plus outer layers 1 and 2; VOI_I1_, tumor minus inner layer 1; VOI_I2_, tumor minus inner layers 1 and 2). In addition, qualitative CT features, including peritumoral enhancement and tumor margin, were assessed. Qualitative and quantitative CT features were compared between HCC patients with and without MVI. Diagnostic performance of DECT parameters of layers and VOIs was assessed using receiver operating characteristic curve analysis.

**Results:**

A total of 36 patients (24 men, mean age 59.9 ± 8.5 years) with MVI (*n* = 14) and without MVI (*n* = 22) were included. HCCs with MVI showed significantly higher NICs of outer layer 1, outer layer 2, VOI_O1_, and VOI_O2_ than those without MVI (*P* = 0.01, 0.04, 0.02, 0.02, respectively). Among the NICs of layers and VOIs, the highest area under the curve was obtained in outer layer 1 (0.747). Qualitative features, including peritumoral enhancement and tumor margin, and the mean CT attenuation of each layer and each VOI were not significantly different between HCCs with and without MVI (both *P* >  0.05).

**Conclusions:**

Volumetric iodine quantification of peritumoral and intratumoral regions in arterial phase using DECT may help predict the MVI of HCC.

## Background

Hepatocellular carcinoma (HCC) is the most common primary liver cancer and is the second leading cause of cancer-related deaths in the Asia-Pacific region [1]. Liver resection and liver transplantation are the first-line curative treatments for eligible patients, but recurrence after surgical treatment is frequent. Previous studies have reported a 5-year recurrence rate of 25% after liver transplantation and 70% after liver resection [2, 3]. Vascular invasion is a prognostic factor for predicting recurrence and overall survival [4]. In particular, microvascular invasion (MVI) is known to be responsible for early recurrence within the first 2 years after curative treatments [5]. Thus, we may be able to identify patients at risk of developing early recurrence if we can preoperatively diagnose MVI.

Previous studies reported that several imaging features of the tumor border on preoperative imaging were associated with MVI of HCC. These features include non-smooth tumor margins, peritumoral enhancement in the arterial phase, peritumoral hypointensity in the hepatobiliary phase, and radiogenomic algorithm based on the association between imaging features (internal arteries and hypoattenuating halos) [6–10]. This is reasonable because, in general, a peripheral portion of many malignant tumors may have the most proliferative or aggressive biological behavior for invading adjacent parenchyma. However, preoperative MVI diagnosis is challenging, and the imaging findings and definition of “peri-” tumoral tissue are subjective and highly reader-dependent. Therefore, if we can reduce the variation and quantify the enhancement of peritumoral tissue, it would be helpful for improving the diagnostic performance of the arterial phase to identify MVI in HCC. Given that material decomposition and iodine quantification by dual-energy CT have been used for many oncologic applications, such as differentiating tumor types and assessing the treatment response of the tumor [11–14], we surmised that iodine concentration could be used for the quantitative assessment of peritumoral enhancement of HCC.

Therefore, the purpose of this study was to identify the potential diagnostic value of iodine quantification in the peritumoral region of HCC by volumetric tumor segmentation with dual-energy computed tomography (DECT) for identifying MVI.

## Methods

The institutional review board of our institution approved this retrospective study and waived the requirement for informed consent.

### Study population

We retrospectively searched our radiology database system between April 2017 and December 2019 to identify eligible patients according to the following criteria: (a) underwent four-phase liver CT at dual-source dual-energy CT (SOMATOM Force; Siemens Healthineers, Forchheim, Germany); (b) underwent hepatic resection within 4 weeks after CT; and (c) surgically confirmed single, treatment-naïve HCC. The exclusion criteria were as follows: (a) preoperative local treatment for the index tumor; (b) < 1 cm, which may increase registration error; and (c) definite macrovascular invasion.

### Histopathology

All surgical specimens were examined by experienced pathologists. The histologic parameters included size, number, Edmondson-Steiner grade, and MVI status of the resected tumor. MVI was defined as the presence of tumor cells in the portal vein, hepatic vein, or a large capsular vessel of the surrounding hepatic tissue lined by the endothelium that was visible only on microscopy. Additionally, the histological grade of fibrosis in the background liver parenchyma was reported on the basis of the staging system for chronic hepatitis by the Korean Study Group for the Pathology of Digestive Diseases (0, no fibrosis; 1, portal fibrosis; 2, periportal fibrosis; 3, septal fibrosis; 4, cirrhosis) [15].

### CT protocol

Liver CT consisted of precontrast, arterial, portal venous, and equilibrium phases. Precontrast, portal venous, and equilibrium phase images were obtained using 90-kVp tube energy before, 70 s, and 180 s after contrast media administration (iobitridol 350 mgI/mL, Xenetix®, Guerbet, France) with weight-based dosing (1.6 mL/kg).

The arterial phase was scanned using dual-energy 17 s after the attenuation of the abdominal aorta reached 80 Hounsfield unit (HU) at 100 kVp, using the care bolus technique of the vendor. For arterial phase dual-energy scanning, a tube potential pair of 80/150 kV with a tin filter was used. The quality reference effective mAs was set to 250 mAs for the 80-kV tube and 125 mAs for the 150-kV tube. Detector configuration, gantry rotation time, and pitch were 192 × 0.6 mm, 0.5 s, and 0.6, respectively. Images were reconstructed with semi-smooth quantitative body kernels in all phases.

### Quantitative imaging analysis

**Fig. 1:**
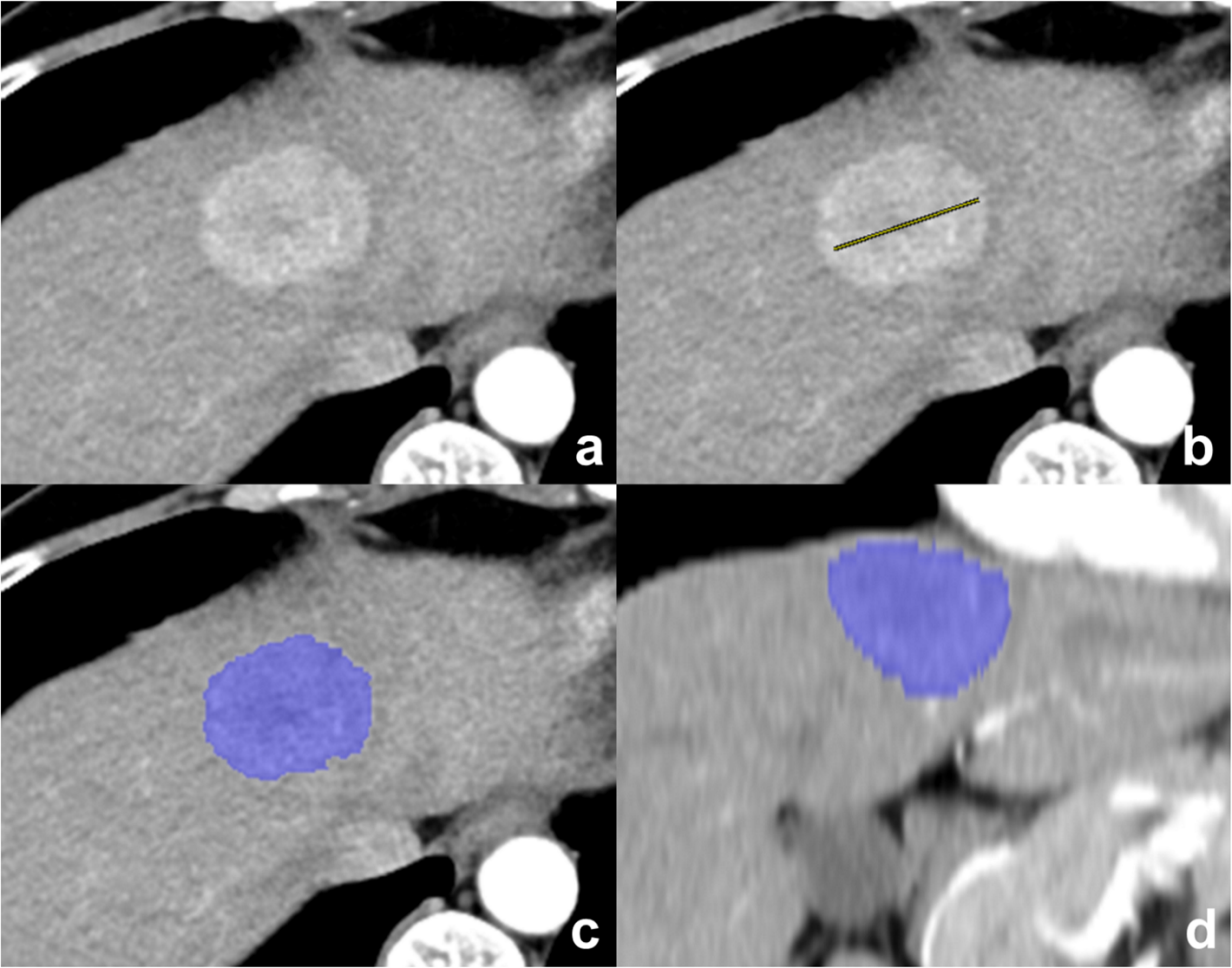
Semiautomatic volumetric segmentation of the hepatocellular carcinoma (HCC). **a** HCC demonstrated in the mixed-energy images. **b** Readers manually draw a line across the maximum dimension of the tumor. **c** The software automatically segments the entire tumor volume. **d** Coronal reconstructed image shows the volumetric tumor segmented in three dimensions

The 80- and 150-kV images of the arterial phase were imported into the prototype software (eXamine: DE Tumor Segmentation, Siemens Healthineers, Forchheim, Germany). The software automatically creates three image sets: the arterial phase of the blended images with a mixed ratio of 0.6 (60% 80 kV and 40% tin-filtered 150 kV), virtual noncontrast, and material density iodine images. Image analysis was performed in mixed-volume images. Two fellowship-trained body radiologists (T.M.K. and S.J.P., 6 years of liver imaging experience) identified the HCC in the mixed-volume images and independently drew a region of interest (ROI) across the maximum dimension of the tumor in any plane (axial, coronal, or sagittal plane) (Fig. 1). Then the software automatically segmented the entire tumor volume and calculated the tumor volume and maximal diameter. In addition, the following were displayed: the mean HU of mixed-energy images, total iodine concentration, and vital iodine concentration. The total iodine concentration refers to the iodine per unit volume (in milligrams per milliliter) in the entire ROI, while vital iodine concentration refers to the iodine per unit volume in the only enhancing portion within the ROI. The tumor margin is manually edited if the tumor is not correctly segmented at once, by evaluating portal venous or equilibrium phase images. The slice-by-slice manual segmentation of the volumetric HCCs were also performed to compare with HCCs obtained by semiautomatic segmentation.

**Fig. 2:**
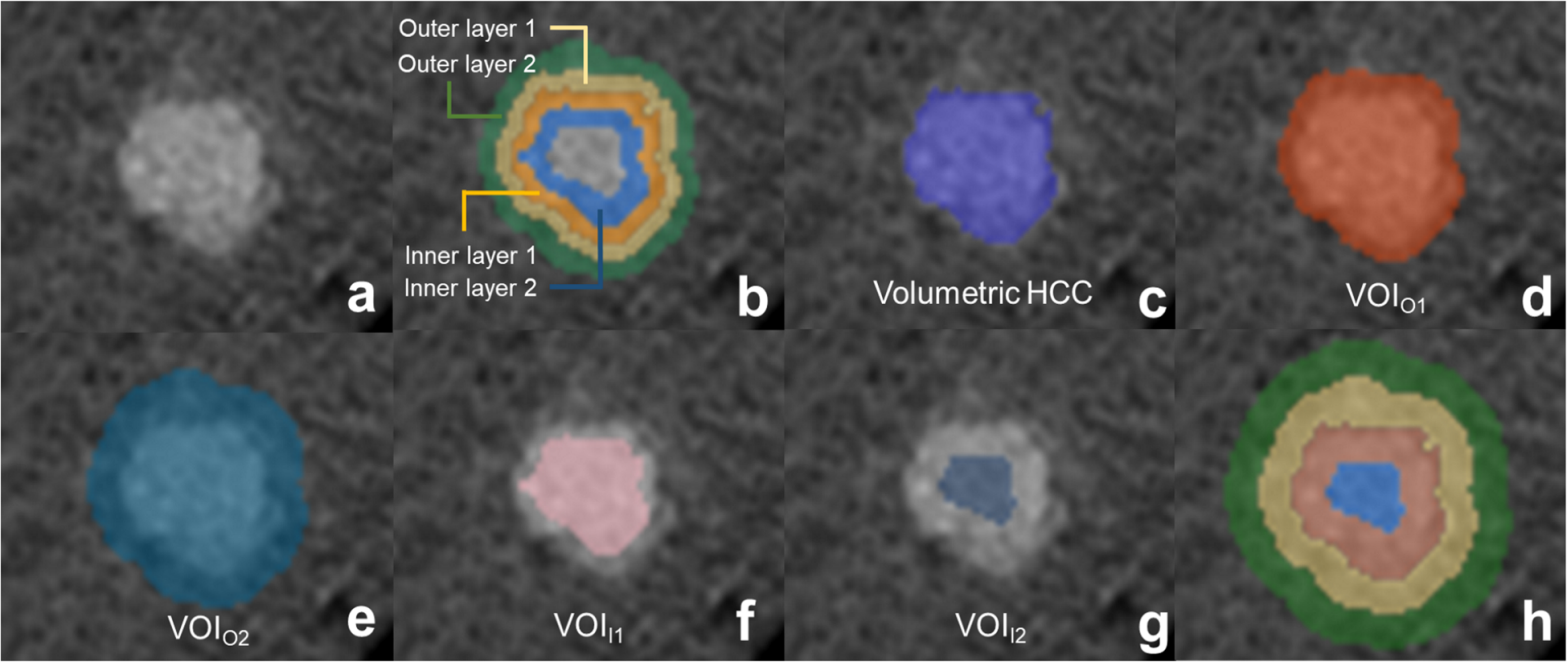
Representative images of the layers and volume of interest (VOIs). **a** Original mixed-energy images. **b** Layer-by-layer analysis for two peritumoral layers (outer layer 1 [yellow], outer layer 2 [green]) and two intratumoral layers (inner layer 1 [orange], inner layer 2 [blue]) with a 2-mm layer thickness. **c** VOI of the tumor. **d** VOI_O1_, tumor plus outer layer 1. **e** VOI_O2_, tumor plus outer layers 1 and 2. **f** VOI_I1_, tumor minus inner layer 1. **g** VOI_I2_, tumor minus inner layers 1 and 2. **h** The aforementioned analysis performed in the same manner with a 4-mm layer thickness

Subsequently, two intratumoral and two peritumoral layers from the tumor border were automatically generated using the peeling function with a layer thickness of 2 mm (outer layer 1, peritumoral region up to 2 mm from tumor margin; outer layer 2, peritumoral region from 2 mm to 4 mm from the tumor margin; inner layer 1, intratumoral region up to 2 mm from the tumor margin; inner layer 2, intratumoral region from 2 mm to 4 mm from the tumor margin). In addition, five VOIs were generated with different volume coverage (tumor itself; VOI_O1_, tumor plus outer layer 1; VOI_O2_, tumor plus outer layers 1 and 2; VOI_I1_, tumor minus inner layer 1; VOI_I2_, tumor minus inner layers 1 and 2) (Fig. 2). For the layers and VOIs outside the tumor, large vessels and areas outside the liver are manually removed.

An ROI was manually drawn in the aorta to calculate the normalized iodine concentration (NIC) to minimize intersubject variations. The software estimates the NIC by dividing vital iodine concentration of the ROI by the iodine concentration in the aorta. Additionally, the software automatically performs segmentation of the entire liver parenchyma to evaluate lesion-to-normal parenchyma ratios of mean attenuation by dividing the mean attenuation of the ROI into those of the normal liver parenchyma.

One radiologist (T.M.K., 6 years of liver imaging experience) blinded to the histologic findings performed the measurement. Additional measurements were performed in separate session by same radiologist (T.M.K.) at least 2 weeks apart and in one session by another radiologist (S.J.P., 6 years of liver imaging experience) to evaluate intraobserver and interobserver reliability, respectively. In addition, the aforementioned quantitative analysis was performed in the same manner but with different layer thicknesses (4 mm) to evaluate the effect of different peritumoral or intratumoral areas.

### Qualitative imaging analysis

Two fellowship-trained body radiologists (T.M.K. and S.J.P., 6 years of liver imaging experience), who were blinded to the clinical and pathological data, independently assessed the images to evaluate the following qualitative features for MVI: (a) presence of peritumoral enhancement, which was defined as the existence of a detectable enhancing portion adjacent to the tumor border in the arterial phase, later becoming isoattenuation in the equilibrium phase [16, 17], and (b) tumor margins, whether they were smooth margins (defined as nodular tumors in all planes) or non-smooth margins (defined as nonnodular tumors in all planes) [16, 17]. All HCCs were evaluated in each dynamic phase using different planes (axial, coronal, and sagittal planes). Disagreements were resolved by consensus. One reader (T.M.K.) repeated the abovementioned assessment in the same manner after 2 weeks to minimize the memory effect to evaluate intraobserver agreement.

### Outcomes

Our primary end point was to evaluate the association between the NIC of peritumoral regions on DECT and MVI in HCC. Secondary end points were as follows: (a) to compare the diagnostic performance of peritumoral NIC with qualitative analysis by a human observer for identifying MVI, (b) to compare the diagnostic performance of peritumoral NIC with peritumoral mean attenuation for identifying MVI, and (c) to investigate the effect of different peritumoral areas (2 mm vs. 4 mm) on the diagnostic performance of MVI.

### Statistical analysis

To compare parameters between the HCCs with and without MVI, continuous data were assessed using the Mann-Whitney test, while categorical variables were analyzed using the chi-square test. Additionally, to investigate the association between subjective peritumoral enhancement and DECT parameters of peritumoral layers, the mean attenuation and layer-to-normal parenchyma ratio of mean attenuation of peritumoral layers were compared between the groups with absent and present peritumoral enhancement. A receiver operating characteristic (ROC) curve analysis was performed using quantitative parameters of layers and VOIs and qualitative parameters to determine the diagnostic performance for the prediction of MVI. Areas under the ROC curves (AUCs) with 95% confidence intervals (CIs) were calculated for the significant CT parameters, and a comparison analysis of AUCs was performed. Variables with *P* < 0.10 in univariate analysis were applied to binary logistic regression analysis. Continuous variables were converted to categorical variables with optimal cutoff values to perform multivariate analysis. Among the NIC of multiple layers and VOIs, only one layer or VOI that showed the highest AUC was included in the multivariate analysis to avoid multicollinearity of parameters.

Intraobserver and interobserver agreements were evaluated using intraclass coefficient class (ICC) and Cohen κ-statistics. The agreements of DECT parameters in volumetric HCCs between manual and semiautomatic segmentation, and agreements of maximal diameter of HCCs obtained in preoperative CT by both segmentation techniques and histopathology were also evaluated using ICC. The strength of agreement via ICC and κ values < 0.4, 0.4–0.6, 0.6–0.8, and >  0.8 were categorized poor, moderate, good, and excellent agreement, respectively. Statistical analyses were performed using a statistical software package (SPSS version 23, SPSS, Inc., Chicago, IL). Differences of *P* < 0.05 were considered statistically significant.

## Results

### Patient characteristics

A total of 47 patients with HCC underwent preoperative liver CT within 4 weeks of hepatic resection. Among them, 11 patients were excluded because of preoperative transarterial chemoembolization (*n* = 7), small size (< 1 cm) of the HCC (*n* = 2), and macrovascular invasion (n = 2). Finally, 36 patients (men/women = 24:12, mean age 59.9 ± 8.5 years) with MVI (*n* = 14) and without MVI (*n* = 22) were included. The median tumor size was 2.7 cm (range, 1.2–4.5 cm). The surgical procedures included segmentectomy (*n* = 23), right anterior sectionectomy (*n* = 1), left lateral sectionectomy (*n* = 3), right hemihepatectomy (*n* = 2), left hemihepatectomy (*n* = 3), and total hepatectomy for transplantation (*n* = 4). The mean time between dual-energy CT and surgery was 16 days (range, 1–30 days). The detailed information of the study group is summarized in Table 1.

**Table 1:** Clinicopathological characteristics of the patients

Characteristics	MVI (−) (*n* = 22, %)	MVI (+) (*n* = 14, %)	*P*-value**
Age (years)			
Median (range)	61.5 (35–74)	56.5 (46–73)	0.4
Sex			
Men	14 (63.6)	10 (71.4)	0.6
Women	8 (36.4)	4 (28.6)	
Staging of hepatic fibrosis^a^			
0	1	0	0.6
1	1	1	
2	5	3	
3	6	3	
4	9	7	
Underlying liver disease			
Hepatitis B	16 (72.7)	12 (85.7)	0.4
Hepatitis C	2 (9.1)	2 (14.3)	
Alcoholism	2 (9.1)	0 (0)	
Non B Non C	2 (9.1)	0 (0)	
Edmondson-Steiner grade of HCC			
I	7	1	0.06
II	12	7	
III	3	6	
AFP level (ng/ml)			
Median (range)	3.02 (0.9–1387.3)	8.89 (2.11–2022.37)	0.03
PIVKA-II level (mAU/ml)			
Median (range)	32 (16–827)	66 (0.74–6168)	0.09

*Abbreviations*: *MVI* microvascular invasion, *AFP* Alpha-fetoprotein, *PIVKA-II* Protein induced by vitamin K absence or antagonist-II, *SD* standard deviation

^a^A staging system for chronic hepatitis by the Korean Study Group for the Pathology of Digestive Diseases was used (0, no fibrosis; 1, portal fibrosis; 2, periportal fibrosis, 3, septal fibrosis; 4, cirrhosis)

** Continuous data were assessed using Mann-Whitney test while categorical variables were analyzed using chi square test. Staging of hepatic fibrosis, underlying liver disease, Edmondson-Steiner grade of HCC were compared as follows; stage 4 vs. stage 0–3, hepatitis B vs. others, grade III vs. grade I-II

Between the two groups with and without MVI, no significant difference was observed in age, sex, histologic grade of hepatic fibrosis, underlying liver disease, and Edmondson-Steiner grade of HCCs (*P* > 0.05, Table 1). Tumor size did not differ between the two groups with and without MVI (3 ± 1.3 cm vs. 2.5 ± 0.9 cm, *P* = 0.17). The group with MVI showed significantly higher serum alpha-fetoprotein (AFP) levels (median 8.9 ng/mL, range 2.1–2022.4 ng/mL) compared with those without MVI (median 3.0 ng/mL, range 0.9–1387.3 ng/mL, *P* = 0.03).

### Comparisons of volumetric DECT parameters between groups with and without MVI

The NICs of outer layer 1, outer layer 2, VOI_O1_, and VOI_O2_ were significantly higher in the MVI group than in the MVI absent group (*P* < 0.05 for all, Table 2). Other layers and VOIs showed no significant differences in NIC values between the two groups (*P* > 0.05).

**Table 2:** The normalized iodine concentrations of peritumoral and intratumoral regions between MVI absent and MVI present groups

Layer thickness	Region*	NIC (mg/ml)		
		MVI(−) (*n* = 22)	MVI(+) (*n* = 14)	*P*-value
2 mm	Outer layer 1	0.07 ± 0.03	0.10 ± 0.03	0.01
	Outer layer 2	0.05 ± 0.03	0.07 ± 0.03	0.04
	Inner layer 1	0.12 ± 0.07	0.16 ± 0.04	0.10
	Inner layer 2	0.15 ± 0.08	0.19 ± 0.07	0.17
	VOI_O1_	0.11 ± 0.05	0.14 ± 0.03	0.02
	VOI_O2_	0.09 ± 0.04	0.12 ± 0.03	0.02
	VOI_I1_	0.15 ± 0.08	0.19 ± 0.07	0.19

*Abbreviations*: *MVI* microvascular invasion, *NIC* normalized iodine concentration, *VOI* volume of interest

Values are presented in means ± standard deviations

*Data which was obtained in part of the patient group was excluded (VOI_I2_, *n* = 33)

For entire volumetric HCCs, quantitative parameters including total volume, maximal diameter, mean HU in mixed-energy images, lesion-to-normal parenchyma ratio of mean attenuation value, total iodine concentration, and NIC showed no difference between HCCs with and without MVI (*P* > 0.05).

### Diagnostic performance of volumetric DECT parameters versus qualitative features for identifying MVI

**Fig. 3:**
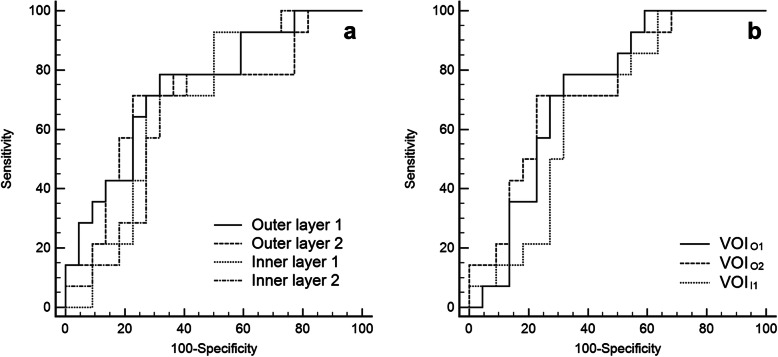
Receiver operating characteristic curves to evaluate the diagnostic performance of normalized iodine concentrations of peritumoral regions to identify microvascular invasion in (**a**) layers and (**b**) volume of interests

The AUCs of NICs of layers and VOIs ranged from 0.67 to 0.75 (Table 3, Fig. 3). The largest AUC was obtained in the NIC of outer layer 1 (0.75). A sensitivity of 78.6% and specificity of 68.2% were obtained at cutoff values of 0.082. There were no significant differences between areas in a pairwise comparison of ROC curves among layers and VOIs (all *P* > 0.05).

**Table 3:** The diagnostic performance of normalized iodine concentrations (NICs) of peritumoral and intratumoral regions and qualitative features for predicting MVI

	NIC*							Qualitative features	
	Outer layer 1	Outer layer 2	Inner layer 1	Inner layer 2	VOI_O1_	VOI_O2_	VOI_I1_	Peritumoral enhancement	Non-smooth tumor margins
AUC	0.75	0.71	0.70	0.68	0.73	0.74	0.67	0.64	0.63
95% CI	0.58–0.91	0.53–0.89	0.52–0.87	0.50–0.85	0.57–0.90	0.58–0.91	0.49–0.85	0.45–0.83	0.44–0.82

*Abbreviations*: *AUC* area under the ROC curve, *CI* confidence interval, *VOI* volume of interest

* Data which was obtained in part of the patient group was excluded (VOI_I2_, *n* = 33)

Peritumoral enhancement of HCCs was present in 36.4% (8/22) of HCCs without MVI and in 64.3% (9/14) of HCCs with MVI. The MVI absent group showed non-smooth tumor margins in 45.5% (10/22), while the MVI present group showed 71.4% (10/14). There were no significant differences between the groups with and without MVI in peritumoral enhancement and non-smooth tumor margins (*P* = 0.10, 0.13). The AUCs of peritumoral enhancement and non-smooth tumor margin were 0.64 (95% CI 0.45–0.83) and 0.63 (95% CI 0.44–0.82), respectively. The AUCs of the NICs and qualitative image features showed no significant differences (*P* > 0.05).

### Multivariate analysis of clinicopathological findings and peritumoral iodine concentration for identifying MVI

**Fig. 4:**
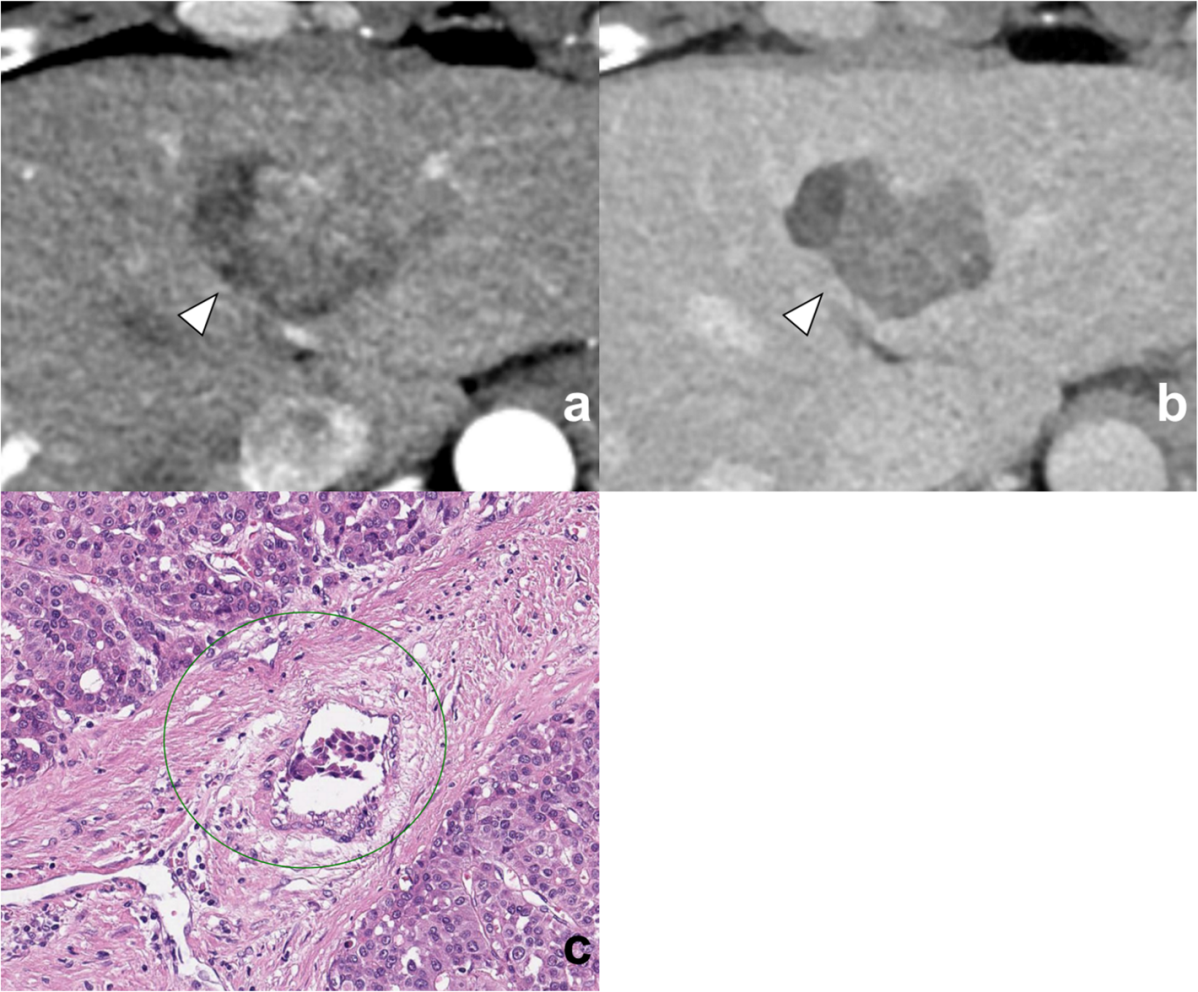
A 54-year-old man with hepatocellular carcinoma with microvascular invasion (MVI). **a** No definite peritumoral enhancement found in the arterial phase compared with **b** equilibrium phase images. However, the NIC of the peritumoral layer with a 2-mm distance from the tumor margin is 0.096 mg/mL, which is higher than the cutoff value for predicting MVI of 0.082 mg/mL. **c** Microscopic evaluation reveals a tumor cells (circle) within the small vessel (hematoxylin-eosin stain, original magnification: × 20 and × 20)

**Fig. 5:**
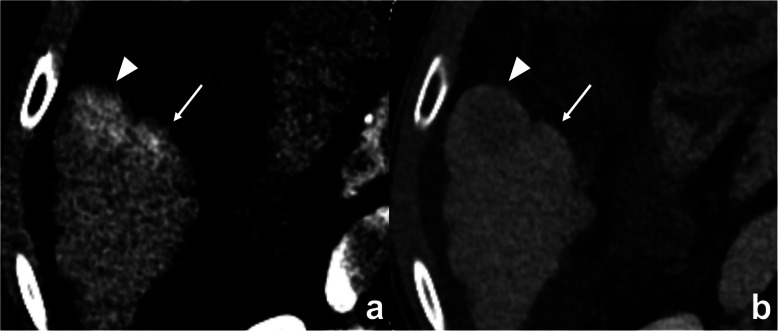
A 64-year-old man with hepatocellular carcinoma (HCC) without microvascular invasion (MVI). **a** Enhancing area found in the peritumoral area (arrows) adjacent to the HCC (arrowheads) in the arterial phase, which became **b** isoattenuation in the equilibrium phase. However, the NIC of the peritumoral layer with a 2-mm distance from the tumor margin is 0.03 mg/mL, lower than 0.082 mg/mL, which is the cutoff value for identifying MVI

In univariate analysis, the MVI group showed more frequent abnormal protein induced by vitamin K absence or antagonist-II (PIVKA-II) level (*P* = 0.05), larger maximal diameter (*P* = 0.04), and higher NIC of outer layer 1 (*P* = 0.02) than the MVI absent group. In multivariate analysis, NIC of outer layer 1 was the only independent factor for predicting MVI, with an odds ratio of 7.14 (*P* = 0.04, Figs. 4 and 5). The serum PIVKA-II level, Edmondson-Steiner grade, and maximal diameter were not significant predictive factors in both sets of multivariate regression analyses (*P* > 0.05, Table 4).

**Table 4:** Univariate and multivariate analyses of clinical and CT parameters in predicting MVI

Parameters	Univariate analysis		Multivariate analysis	
	Odds ratio (95% CI)	*P*-value	Odds ratio (95% CI)	*P*-value
Age (> 60 years)	0.63 (0.16–2.41)	0.50		
Sex (Female)	0.70 (0.16–2.98)	0.63		
Staging of hepatic fibrosis (Stage 4)	1.44 (0.38–5.57)	0.59		
AFP (> 8.7 ng/ml)	2.67 (0.65–10.88)	0.17		
PIVKA-II (> 40 mAU/mL)	4.38 (1.03–18.63)	0.05	2.71 (0.47–15.58)	0.26
Edmondson-Steiner grade of HCC (III)	4.75 (0.95–23.85)	0.06	5.64 (0.67–47.25)	0.11
Total volume of HCC (> 4.5 ml)	2.16 (0.54–8.57)	0.27		
Maximal diameter of HCC (> 32 mm)	4.53 (1.06–19.41)	0.04	4.04 (0.67–24.37)	0.13
Peritumoral enhancement	3.15 (0.78–12.73)	0.11		
Nonsmooth tumor margin	3.00 (0.72–12.55)	0.13		
NIC of outer layer 1 (> 0.082 mg/ml)	6.42 (1.37–30.05)	0.02	7.14 (1.12–45.40)	0.04

*Abbreviations*: *MVI* microvascular invasion, *AFP* Alpha-fetoprotein, *PIVKA-II* Protein induced by vitamin K absence or antagonist-II, *NIC* normalized iodine concentration, *VOI* volume of interest, *CI* confidence interval

### Relationship between peritumoral enhancement and volumetric DECT parameters of the peritumoral layers

Layer-to-normal parenchyma ratio of mean HU values was significantly higher in outer layer 2, in both 2-mm and 4-mm layer thickness, in HCCs with peritumoral enhancement than in those in HCCs without peritumoral enhancement (*P* = 0.03, *P* = 0.04, respectively). However, the mean HU and NIC showed no significant difference between the two groups (*P* > 0.05, Supplementary Table 1, Additional file 1).

### Diagnostic performance of peritumoral NICs versus peritumoral CT attenuations for identifying MVI

The mean attenuations of all layers and VOIs showed no significant difference between the two groups (*P* > 0.05, Supplementary Table 2, Additional file 2). The AUCs of the mean attenuations of layers and VOIs ranged from 0.56 to 0.66. They were not significantly different from the AUCs of NICs in pairwise comparisons (all *P* > 0.05).

### Effect of layer thickness

In the 4-mm layer thickness, the NICs of outer layer 1, VOI_O1_, and VOI_O2_ were also significantly higher in the MVI group than in the MVI absent group (all *P* < 0.05, Supplementary Table 3, Additional file 3). The AUCs of the NICs ranged from 0.69 to 0.75. The largest AUC was obtained for VOI_O2_ (AUC 0.75). There were no significant differences between areas in a pairwise comparison of ROC curves, including the values obtained in 2-mm layer thickness (all *P* > 0.05).

### Comparison of volumetric HCC between manual segmentation and semiautomatic segmentation

The agreements of DECT parameters of volumetric HCCs between two segmentation techniques were excellent (*ICC* = 0.97–1.0, Supplementary Table 4, Additional file 4). Maximal diameter of HCCs in histopathology (26.7 ± 10.8 mm) showed excellent correlation with those of HCCs in preoperative CT by both manual (27.2 ± 9.8 mm, *ICC* = 0.94) and semiautomatic (28.3 ± 10.7 mm, *ICC* = 0.91) segmentation methods.

### Intra- and interobserver agreements

Interobserver and intraobserver agreements were excellent in all measured quantitative parameters (*ICC* = 0.81–1.0, 0.85–1.0). In qualitative parameters, interobserver and intraobserver agreements were good to excellent and excellent, respectively (κ = 0.67–0.71, 0.78–0.89, Supplementary Table 5, Additional file 5).

## Discussion

Our study demonstrated that the NIC of the peritumoral zones and intratumoral zones of HCCs measured by DECT was useful for the prediction of MVI. In more detail, outer layer 1 and outer layer 2, which corresponded to the peritumoral layers, showed significantly higher NIC in the MVI present group than in the MVI absent group. Interestingly, the combination of HCCs and peritumoral regions (VOI_O1_, VOI_O2_) also showed different NIC values between the MVI present and absent groups with comparable AUCs to those of peritumoral regions only. This might imply that MVI status could be predicted by analyzing NICs of both intratumoral and peritumoral regions as well as peritumoral regions only. We also found that the NIC of the peritumoral region up to 2 mm from the tumor margin was an independent predictive factor for MVI in multivariate analysis. Yang et al. indicated that the NICs of HCCs with MVI were significantly higher than those of HCCs without MVI [18]. However, our results showed no significant difference in the NICs of HCCs between the two groups. This discrepancy may have contributed to the difference in the way ROI was placed. A previous study drew the ROI with a diameter of half of the tumor size in one axial image, whereas we segmented the whole volume of the HCCs in three dimensions, which better reflects the nature of the tumor.

Peritumoral enhancement is usually defined as grossly contrast material enhancement outside of the tumor border at the arterial phase that becomes isointense with background liver parenchyma in the later dynamic phase images [19]. This imaging finding probably relates to the known hypothesis of hemodynamic perfusion changes existing in compensatory arterial hyperperfusion, which can occur in the region of decreased portal flow caused by minute portal branch occlusion because of tumor thrombi, assuming that the draining veins of HCCs are usually portal venules [20]. Until now, there has been controversy as to whether there is a correlation between peritumoral enhancement in CT and MVI of HCCs. Previous studies have demonstrated that peritumoral enhancement significantly increases the risk of MVI, and the area of peritumoral hemodynamic change in HCC patients with microscopic portal invasion was significantly larger than those without it in combined CT hepatic arteriography and CT arterioportography [21, 22]. However, another study by Chou et al. described peritumoral enhancement in CT was not a significant risk factor for MVI [16]. One of the potential reasons for these discrepancies among the previous studies could be the qualitative assessment of peritumoral enhancement, ROI measurement of attenuation coefficient, and heterogeneous scanning parameters. Although qualitative image findings such as peritumoral enhancement and non-smooth tumor margins showed similar AUCs compared with those of NICs in our study, there were no significant factors in univariate analysis for predicting MVI, in contrast to previous studies [7, 9, 16, 17, 22]. The number of patients might be insufficient to evaluate these multiple qualitative imaging features. However, the NIC of the peritumoral layer was an independent predictive factor for MVI in this setting, which suggests an additional value for MVI prediction in preoperative imaging. The diagnostic accuracy of our study (AUC 0.67–0.75, sensitivity 78.6%, specificity 68.2%) was comparable to that of a recent study that evaluated peritumoral enhancement qualitatively in CT (accuracy 74.3%) [22].

In our study, despite the attenuation coefficients of peritumoral areas showed lower AUCs (0.56–0.66) than peritumoral NICs (0.67–0.75), there was no statistical difference. It might be contributed to the small study population. However, in contrast to peritumoral NICs, there were no significant factors for the prediction of MVI in peritumoral attenuation coefficients. A previous study revealed that dual-energy CT demonstrated a linear relationship with low relative error (less than 10%) between measured and actual iodine concentrations in an in vitro experiment [23]. Based on our study results, we suggest that the iodine concentration calculated using dual-energy CT may reflect peritumoral perfusional changes caused by microcirculation of the blood flow more accurately than attenuation measurements.

A recent study demonstrated radiomic analysis of contrast-enhanced CT for predicting MVI [22]. They integrated the radiomic features of peritumoral and intratumoral region with clinical factors and radiographic features, and this predictive model achieved high accuracy in estimating MVI with an AUC of 0.89. In this study, they found the radiomic features of peritumoral zone at 5 mm distance from the tumor surface differ according to the presence of MVI. Our results are in concordance with this study, in that the quantitative characteristics of the peritumoral area were different depending on the presence or absence of MVI. One thing we should point out is, the patients in our study have variable degree of liver fibrosis. According to previous literatures, with hepatic fibrogenesis, microcirculatory changes results in increased total hepatic resistance with altered portal venous blood flow, compensated by increased hepatic arterial flow [24, 25]. Liver perfusion decreases while arterial perfusion increases in patients with cirrhosis, and correlates with severity [26]. The presence of liver cirrhosis (stage 4 of hepatic fibrosis) were not significantly different between MVI present and absent group, but heterogeneity of fibrosis degree might change the enhancement characteristics of the peritumoral parenchyma and subsequently affect both quantitative and qualitative analysis of peritumoral region.

The main issue of our study was how to correctly draw the tumor boundary. The initial process for tumor segmentation is the most important step because it affects all of the peritumoral layers and intratumoral layers in the following process. We used a dedicated software prototype (eXamine: “DE Tumor Segmentation”) that allows for a refined volumetric semiautomatic segmentation of the entire tumor and a respective evaluation of spectral data. This application is designed to evaluate different parameters of a tumor scanned with Siemens DECT technology using an iodine contrast agent. It also provides semiautomatic segmentation of the tumor peel datasets. Once the user selects the number of peels in the prototype, the user obtains the respective number of peels to the outside and inside of the original tumor automatically and a respective analysis of the different layers from a spectral perspective. We additionally performed manual segmentation of HCCs to evaluate the performance of semiautomatic segmentation, and found high agreement between the DECT parameters of HCCs in both segmentation techniques (ICC 0.97–1.0). One remarkable observation in our study is that we found strong correlations between the macroscopic diameter of the surgical tumors and the maximal diameter of HCCs obtained by semiautomatic segmentation (ICC 0.91), as well as manual segmentation (ICC 0.94). These results suggest, although we had to edit the tumor margins manually in a few HCCs with ill-defined margins, semiautomatic segmentation technique used in the current study can perform tumor segmentation accurately and efficiently. It minimized the hand-related artifacts and showed excellent intra- and interobserver agreements. The manual drawing in each slice was also accurate and simple but takes a lot of time and effort. Our study is in concordance with previous studies that demonstrated good correlation between manual and semiautomatic segmentation, while latter technique can reduce the time for measurement and show excellent accuracy and repeatability [27–30]. This technique can also be applied to the segmentation of tumors other than HCCs. The layer thickness (2 mm or 4 mm) did not affect the diagnostic performance in our study. This result suggests that perfusional change occurs in the peritumoral region within a distance of at least 8 mm from the tumor margin.

This study has some limitations. First, this retrospective study was performed in a single center with a small number of patients. Additionally, only surgically confirmed HCCs were included in this study. A potential selection bias might have occurred. However, because MVI can only be confirmed by pathology, strict inclusion criteria are inevitable. Second, tumors less than 1 cm were excluded from our study because of the potential measurement error. Although it was not intended, all HCCs of the patients were less than 5 cm in maximal diameter. Therefore, our results cannot be generalized to small (< 1 cm) or large (> 5 cm) HCCs. Third, various degree of liver fibrosis in our study population may affect the enhancement characteristics of peritumoral region and results of our study. A larger study with subgroup analysis according to the fibrosis degree would be helpful to decrease this bias. Fourth, the software used in our study was only available in dual-source dual-energy CT. Other dual-energy techniques, such as rapid kVp switching or dual-layer spectral CT, were not used in our study. Further studies using other dual-energy CT vendors are warranted.

## Conclusion

In conclusion, the volumetric iodine quantification of peritumoral regions in arterial phase may be useful for predicting MVI in preoperative dual-energy CT.

## Supplementary information

**Additional file 1: Table S1.** Relationship between peritumoral enhancement and quantitative parameters of peritumoral layers.

**Additional file 2: Table S2.** The Hounsfield units of peritumoral and intratumoral regions between MVI absent and MVI present groups.

**Additional file 3: Table S3.** The normalized iodine concentration (NICs) of peritumoral and intratumoral regions in 4 mm layer thickness between MVI absent and MVI present groups.

**Additional file 4: Table S4.**. Comparison of semiautomatic segmentation and manual segmentation.

**Additional file 5: Table S5.** Intraobserver and interobserver agreements of quantitative and qualitative parameters.

## Data Availability

All data generated or analysed during this study are included in this published article [and its supplementary information files].

## Abbreviations

CT: Computed tomography
DECT: Dual-energy computed tomography
HCC: Hepatocellular carcinoma
MVI: Microvascular invasion
NIC: Normalized iodine concentration
VOI: Volume of interest
