# Supplementary material for: Prediction of microvascular invasion of hepatocellular carcinoma: value of volumetric iodine quantification using preoperative dual-energy computed tomography
Source: Cancer Imaging. 2020 Aug 18;20:60. doi: 10.1186/s40644-020-00338-7 (PMC7433153; doi:10.1186/s40644-020-00338-7)
Supplement: Supplementary file 1 — Additional file 1: Table S1. Relationship between peritumoral enhancement and quantitative parameters of peritumoral layers. [file 40644_2020_338_MOESM1_ESM.docx]

**Supplementary Table 1**. Relationship between peritumoral enhancement and quantitative parameters of peritumoral layers.

| Layer thickness | Region | Parameters | Peritumoral enhancement (-) | Peritumoral enhancement (+) | P-value |  |
| --- | --- | --- | --- | --- | --- | --- |
|  |  |  | (n = 19) | (n = 17) |  | |
| 2 mm | Outer layer 1 | Mean HU | 74.4 ± 10.6 | 80.4 ± 13.6 | 0.21 | |
|  |  | Layer-to-normal parenchyma ratio of mean HU | 1.02 ± 0.08 | 1.08 ± 0.12 | 0.09 | |
|  |  | NIC | 0.08 ± 0.04 | 0.09 ± 0.03 | 0.36 | |
|  | Outer layer 2 | Mean HU | 76.9 ± 12.2 | 85.6 ± 12.9 | 0.07 | |
|  |  | Layer-to-normal parenchyma ratio of mean HU | 1.05 ± 0.12 | 1.15 ± 0.11 | 0.03 | |
|  |  | NIC | 0.06 ± 0.03 | 0.06 ± 0.03 | 0.57 | |
| 4 mm | Outer layer 1 | Mean HU | 74.5 ± 10.2 | 77.6 ± 12.0 | 0.68 | |
|  |  | Layer-to-normal parenchyma ratio of mean HU | 1.02 ± 0.05 | 1.04 ± 0.09 | 0.85 | |
|  |  | NIC | 0.07 ± 0.03 | 0.07 ± 0.03 | 0.53 | |
|  | Outer layer 2 | Mean HU | 75.2 ± 10.2 | 82.1 ± 13.6 | 0.15 | |
|  |  | Layer-to-normal parenchyma ratio of mean HU | 1.03 ± 0.09 | 1.10 ± 0.11 | 0.04 | |
|  |  | NIC | 0.04 ± 0.02 | 0.05 ± 0.03 | 0.66 | |

Abbreviations: VOI, volume of interest; HU, Hounsfield unit; NIC, normalized iodine concentration.
