# Supplementary material for: Prediction of microvascular invasion of hepatocellular carcinoma: value of volumetric iodine quantification using preoperative dual-energy computed tomography
Source: Cancer Imaging. 2020 Aug 18;20:60. doi: 10.1186/s40644-020-00338-7 (PMC7433153; doi:10.1186/s40644-020-00338-7)
Supplement: Supplementary file 2 — Additional file 2: Table S2. The Hounsfield units of peritumoral and intratumoral regions between MVI absent and MVI present groups. [file 40644_2020_338_MOESM2_ESM.docx]

**Supplementary Table 2**. The Hounsfield units of peritumoral and intratumoral regions between MVI absent and MVI present groups.

| Layer thickness | Region | Hounsfield unit | | |
| --- | --- | --- | --- | --- |
|  |  | MVI(-) (n=22) | MVI(+) (n=14) | P-value |
| 2 mm | Outer layer 1 | 74.4 ± 11.6 | 81.8 ± 12.4 | 0.08 |
|  | Outer layer 2 | 78.4 ± 13.4 | 85.1 ± 12.1 | 0.14 |
|  | Inner layer 1 | 97.0 ± 22.9 | 104.8 ± 23.0 | 0.33 |
|  | Inner layer 2 | 105.4 ± 27.7 | 112.4 ± 33.9 | 0.50 |
|  | VOI_O1_ | 90.3 ± 17.5 | 97.1 ± 15.8 | 0.24 |
|  | VOI_O2_ | 84.5 ± 13.1 | 91.7 ± 12.2 | 0.11 |
|  | VOI_I1_ | 105.0 ± 27.8 | 111.4 ± 33.9 | 0.54 |
| 4 mm | Outer layer 1 | 73.4 ± 10.1 | 80.0 ± 11.5 | 0.08 |
|  | Outer layer 2 | 75.7 ± 11.8 | 82.7 ± 12.2 | 0.09 |
|  | Inner layer 1 | 100.0 ± 24.3 | 107.2 ± 25.3 | 0.40 |
|  | VOI_O1_ | 84.5 ± 13.1 | 91.7 ± 12.2 | 0.11 |
|  | VOI_O2_ | 78.9 ± 10.3 | 86.2 ± 11.0 | 0.05 |

Abbreviations: MVI, microvascular invasion; NIC, normalized iodine concentration; VOI, volume of interest.

Values are presented in means ± standard deviations.

* Data which was obtained in part of the patient group was excluded (VOI_I2_ [2mm], n = 33; Inner layer 2 [4mm], n = 33; VOI_I1_ [4mm], n = 33; VOI_I2_ [4mm], n = 14).
