# Supplementary material for: Prediction of microvascular invasion of hepatocellular carcinoma: value of volumetric iodine quantification using preoperative dual-energy computed tomography
Source: Cancer Imaging. 2020 Aug 18;20:60. doi: 10.1186/s40644-020-00338-7 (PMC7433153; doi:10.1186/s40644-020-00338-7)
Supplement: Supplementary file 3 — Additional file 3: Table S3. The normalized iodine concentration (NICs) of peritumoral and intratumoral regions in 4 mm layer thickness between MVI absent and MVI present groups. [file 40644_2020_338_MOESM3_ESM.docx]

**Supplementary Table 3**. The normalized iodine concentration (NICs) of peritumoral and intratumoral regions in 4mm layer thickness between MVI absent and MVI present groups.

| Layer thickness | Region | NIC (mg/ml) | | |
| --- | --- | --- | --- | --- |
|  |  | MVI(-) (n=22) | MVI(+) (n=14) | P-value |
| 4 mm | Outer layer 1 | 0.06 ± 0.03 | 0.08 ± 0.03 | 0.03 |
|  | Outer layer 2 | 0.04 ± 0.02 | 0.06 ± 0.03 | 0.05 |
|  | Inner layer 1 | 0.13 ± 0.07 | 0.17 ± 0.05 | 0.06 |
|  | VOI_O1_ | 0.09 ± 0.04 | 0.12 ± 0.03 | 0.02 |
|  | VOI_O2_ | 0.06 ± 0.03 | 0.09 ± 0.03 | 0.01 |

Abbreviations: MVI, microvascular invasion; NIC, normalized iodine concentration; VOI, volume of interest.

Values are presented in means ± standard deviations.

* Data which was obtained in part of the patient group was excluded (Inner layer 2, n = 33; VOI_I1_, n = 33; VOI_I2_, n = 14).
