# Supplementary material for: Prediction of microvascular invasion of hepatocellular carcinoma: value of volumetric iodine quantification using preoperative dual-energy computed tomography
Source: Cancer Imaging. 2020 Aug 18;20:60. doi: 10.1186/s40644-020-00338-7 (PMC7433153; doi:10.1186/s40644-020-00338-7)
Supplement: Supplementary file 4 — Additional file 4: Table S4.. Comparison of semiautomatic segmentation and manual segmentation. [file 40644_2020_338_MOESM4_ESM.docx]

**Supplementary Table 4.** Comparison of semiautomatic segmentation and manual segmentation.

Abbreviations: DECT, dual-energy computed tomography; HCC, hepatocellular carcinoma; HU, Hounsfield unit; ICC, intra-class correlation coefficients

* Agreements were assessed with intra-class correlation coefficients (ICC).

| DECT Parameters of HCC | Semiautomatic segmentation | Manual segmentation | ICC* | |
| --- | --- | --- | --- | --- |
| Total volume (ml) | 8.8 ± 9.2 | 7.8 ± 7.5 | | 0.968 |
| Maximal diameter (mm) | 28.3 ± 10.7 | 27.2 ± 9.8 | | 0.981 |
| Mean HU (HU) | 102.9 ± 24.5 | 101.5 ± 24.0 | | 0.996 |
| Total iodine concentration (mg/ml) | 1.91 ± 0.82 | 1.89 ± 0.80 | | 0.995 |
| Normalized iodine concentration (mg/ml) | 0.15 ± 0.06 | 0.14 ± 0.06 | | 0.994 |
