# Supplementary material for: Prediction of microvascular invasion of hepatocellular carcinoma: value of volumetric iodine quantification using preoperative dual-energy computed tomography
Source: Cancer Imaging. 2020 Aug 18;20:60. doi: 10.1186/s40644-020-00338-7 (PMC7433153; doi:10.1186/s40644-020-00338-7)
Supplement: Supplementary file 5 — Additional file 5: Table S5. Intraobserver and interobserver agreements of quantitative and qualitative parameters. [file 40644_2020_338_MOESM5_ESM.docx]

**Supplementary Table 4.** Intraobserver and interobserver agreements of quantitative and qualitative parameters.

|  |  | Qualitative features | | DECT parameters of volumetric HCCs | | | | | Mean HU and NIC of layers and VOIs | | | |
| --- | --- | --- | --- | --- | --- | --- | --- | --- | --- | --- | --- | --- |
|  |  |  |  |  |  |  |  |  | 2-mm layer thickness | | 4-mm layer thickness | |
|  |  | Peritumoral enhancement | Non-smooth tumor margins | Total volume (ml) | Maximal diameter (mm) | Mean HU (HU) | Total iodine concentration (mg/ml) | NIC (mg/ml) | Layers | VOIs | Layers | VOIs |
| Semiautomatic segmentation | Inter-observer agreement | .670  (<.001) | .713 (<.001) | .997 (<.001) | .991 (<.001) | .999 (<.001) | .998 (<.001) | .998 (<.001) | .810-.994 (<.001) | .995-.998 (<.001) | .991-.997 (<.001) | .990-.997  (<.001) |
|  | Intra-observer agreement | .888  (<.001) | .775 (<.001) | .999 (<.001) | .995 (<.001) | .999 (<.001) | .999 (<.001) | .998 (<.001) | .849-.995  (<.001) | .994-.999  (<.001) | .993-.998  (<.001) | .992-.999  (<.001) |
| Manual segmentation | Inter-observer agreement |  |  | .985 (<.001) | .977  (<.001) | .997  (<.001) | .994 (<.001) | .996 (<.001) |  |  |  |  |
|  | Intra-observer agreement |  |  | .993 (<.001) | .976  (<.001) | .998  (<.001) | .997 (<.001) | .998 (<.001) |  |  |  |  |

Abbreviations: DECT, dual-energy computed tomography; HCC, hepatocellular carcinoma; HU, Hounsfield unit; NIC, normalized iodine concentration; VOI, volume of interest.

* Agreements regarding categorical and continuous variables were assessed with Cohen’s κ statistics and intra-class correlation coefficients (ICC), respectively
